# Oral Rhizoma Coptis Alkaloids Nanoparticle for Treating Diabetes Through Regulating PI3K/Akt Pathways

**DOI:** 10.3390/pharmaceutics18030349

**Published:** 2026-03-11

**Authors:** Yuejiao Liu, Mengyuan Zhu, Qiaoqiao Su, Maofeng Liu, Zhenyu Zhao, Pengkai Ma

**Affiliations:** 1 NHC Key Lab of Hormones and Development and Tianjin Key Lab of Metabolic Diseases, Tianjin Medical University Chu Hsien-I Memorial Hospital & Institute of Endocrinology, Tianjin 300000, China; 2 School of Chinese Materia Medica, Beijing University of Chinese Medicine, Beijing 100000, China

**Keywords:** type 2 diabetes mellitus, *Rhizoma Coptidis*, alkaloids, nanomedicine, nanoparticle

## Abstract

**Objectives**: Rhizoma Coptidis alkaloids (RCAs) have been proven highly promising in diabetes therapy. However, poor solubility, low bioavailability, and a lack of an effective delivery strategy are major hurdles to improving clinical outcomes. Herein, mPEG-PLGA nanoparticles were employed to deliver RCA orally to enhance anti-diabetic effects. **Methods**: The RCA-loaded nanoparticles (RCA NPs) were prepared using the emulsion solvent diffusion method. The physicochemical properties of RCA NPs were characterized by morphology, particle size, zeta potential, polydispersity index, drug loading, and drug release. Pharmacokinetic and tissue distribution were determined by UPLC-MS/MS. The hypoglycemic effect was evaluated in a type 2 diabetes mouse model. To illustrate potential mechanisms of action, the expression of PI3K/Akt signaling pathway-related genes and their proteins was detected by RT-PCR and Western blot, respectively. **Results**: The prepared RCA NPs were spherical in structure, with a particle size of approximately 145 nm and a sustained drug release profile (approximately 50% within 24 h). Compared with RCAs, RCA NP bioavailability increased approximately 2.2-fold, and the hypoglycemic, hypolipidemic, hepatoprotective, anti-inflammatory effects were significantly improved. The better outcome might be due to upregulation of expression and phosphorylation levels within the IRS1/PI3K/AKT/GLUT4 signal pathway in liver tissues. **Conclusions**: RCA NPs hold great potential for further clinical translation.

## 1. Introduction

With the prevalence of type 2 diabetes mellitus (T2DM), exploring safe and effective hypoglycemic drugs is importance for preventing T2DM and its complications. Though guanidines, glitazones, acylureas, and peptides are the most frequently used drugs in clinical settings, natural medicine might be an alternative and promising treatment strategy, given its satisfactory therapeutic effects, minor adverse effects, and cost-effectiveness [1,2,3].

Rhizoma Coptidis (RC, Chinese name: Huanglian), a traditional herbal medicine, has been used in China for thousands of years to treat diabetes [4]. The vital pharmacodynamic components have been identified as alkaloids, predominantly berberine, epiberberine, palmatine, coptisine, and brucine [5]. Though each of them has been found to possess anti-diabetic activity, studies have shown that total alkaloids exhibited a superior anti-diabetic effect compared with a single component due to a multi-path and multi-target action mode [6,7], which relies on inhibiting glucose absorption in the digestive tract, accelerating glucose uptake and metabolism, protecting islet β cells, promoting insulin secretion, and reducing insulin resistance [8,9]. However, its clinical application has been severely hindered by its low aqueous solubility and poor bioavailability and pharmacokinetics [10]. Therefore, there is an urgent need to develop reliable strategies to overcome RCA-related weaknesses for further clinical application.

Polymeric nanoparticles are a class of nanoscale particles (typically 10–1000 nm) comprising natural or synthetic polymers that exhibit great potential in biomedical applications, especially in drug delivery systems [11,12]. Drugs encapsulated in polymeric nanoparticles benefit from increased bioavailability, prolonged release, reduced dosing, and improved patient compliance. After modification with functional moieties, such as targeting ligands, the nanoparticles can further specifically interact with receptors overexpressed on cells. Moreover, co-delivery of multiple drugs within the same nanocarrier offers advantages over the administration of single-drug nanomedicines or a combination of free drugs at the same dosage [13]. When different drugs act on several targets, they tend to produce a synergistic effect, helping to avoid drug resistance. Polymeric nanoparticles can be prepared from numerous polymer materials, such as poly lactic acid (PLA), poly lactic acid–co-glycolic acid (PLGA), and chitosan. Among them, PLGA is one of the most successful polymer materials used in commercial polymeric nanoparticle formulations due to its well-established preparation technology and reliable quality control [14]. Inspired by these advancements, we employed PLGA to prepare RCA-loaded polymeric nanoparticles, evaluate their pharmacodynamic effects in a T2DM mouse model, and illustrate their potential mechanisms of action. This study would provide a reference for developing a novel RCA formulation to treat T2DM in the clinical setting.

## 2. Materials and Methods

### 2.1. Reagents

*Rhizoma Coptidis* was purchased from Bencaofangyuan Co., Ltd. (Beijing, China). PLGA was bought from Ruixi Biological (Xi’an, China). Reference standards (berberine, epiberberine, palmatine, coptisine, jatrorrhizine, and magnoflorine, purity 99%) were bought from Yuanye Bio-Technology Company (Shanghai, China). Simulated gastric fluid (SGF, USP standard, sterile) and simulated intestinal fluid (SIF, USP standard, sterile) were bought from Beijing Leagene Biotechnology Co., Ltd. (Beijing, China). Biochemical assay kits, including total cholesterol (TC), triglyceride (TG), high/low density lipoprotein cholesterol (H/LDL-C), insulin, interleukin 1 Beta (IL-1β), interleukin 6 (IL-6), and tumor necrosis factor α (TNF-α), were obtained from Biorigin (Beijing) Inc. (Beijing, China). Primary antibodies for IL-1β, IL-6, TNF-α, β-actin, IRS1, p-IRS1, PI3K, p-PI3K, AKT, p-AKT, and GLUT4 were obtained from Abcam (Shanghai) Co., Ltd. (Shanghai, China). Other chemical reagents were purchased from Sigma Aldrich (Shanghai) Co., Ltd. (Shanghai, China).

### 2.2. Animals

C57BL/6J mice (6 weeks old, male, weighing 18–22 g) were purchased from SiPeiFu Biotechnology Co., Ltd. (Beijing, China). Animals were fed in the SPF animal house with free access to food and water. The T2DM mouse model was established using the high-fat diet/streptozotocin method [15]. Mice were first fed a high-fat diet (containing 60% fat) for 4 weeks. And then, they were intraperitoneally administered with streptozotocin (STZ) at a dose of 90 mg·kg^−1^ twice consecutively within 72 h. Blood glucose (BG) was measured using a glucometer (Johnson & Johnson’s, New Brunswick, NJ, USA). The inclusion criterion was a blood glucose level of ≥16.7 mmol·L^−1^. Animal experiments were conducted according to the guidelines and ethics of the Chinese Animal Protection Committee and approved by the Laboratory Animal Care and Use Committee of Beijing University of Chinese Medicine (license number: BUCM-4-2021051005-2120).

### 2.3. Preparation and Chromatographic Characterization of RCA

Rhizoma Coptidis alkaloids (RCAs) were prepared using a method described elsewhere [6]. Briefly, Rhizoma Coptidis powder was added to a 1.5% sulfuric acid solution at a ratio of 1:8 (kg:L) and refluxed under heat for three cycles (40 min per cycle). After filtration, calcium hydroxide was added to adjust the pH to 6.0. After filtration, hydrochloric acid was added to adjust the pH to 2.0. Then, 5% sodium chloride solution was added, and the mixture was refrigerated (4 °C) overnight. Finally, the precipitate was dried at 50 °C to obtain RCA. An HPLC method was established to determine the alkaloids. HPLC detection conditions were as follows: chromatographic column, Agilent EXTEND-C18 column (4.6 mm × 250 mm, 5 μm); mobile phase, acetonitrile and 0.05 mol·L^−1^ KH_2_PO_4_ solution containing 0.4% sodium dodecyl sulfonate (pH 4.0, 45:55); detection wavelength, 276 nm; flow rate, 1.0 mL·min^−1^; column temperature, 30 °C; injection volume, 5 μL.

### 2.4. Preparation of RCA NPs

The RCA-loaded mPEG-PLGA nanoparticles (RCA NPs) were prepared using the emulsion solvent diffusion method [16]. In brief, RCA and mPEG-PLGA were dissolved in a mixed solvent of methanol and dichloromethane (2/1, *v*/*v*) to form an organic phase, and an appropriate amount of emulsifier was dissolved in deionized water to form the water phase. Then, the organic phase was dripped into the water phase under high-speed homogenization to form a coarse emulsion. The coarse emulsion was further homogenized at high pressure using a microfluid homogenizer (Mikrofluidics, Westwood, MA, USA) to obtain a fine emulsion. Next, the mini-emulsion was added to deionized water pre-cooled to 4 °C for solidification. The organic solvent was removed by rotary evaporation, and the free drug was removed after centrifuging at 3000 *g* for 5 min in an ultrafiltration tube (MWCO 10 kDa). Finally, the suspension was freeze-dried to obtain the RCA NPs. Using particle size and drug loading as indexes, the factors influencing nanoparticle preparation were investigated to determine the optimal prescription and preparation process.

### 2.5. Physicochemical Properties of RCA NPs

The lyophilized nanoparticles were weighed precisely and dissolved in methanol. RCA content was determined using the HPLC method. The loading capacity (LC) was calculated with the following formula:

$$\mathrm{LC}\;(100\%)\;=\;\frac{\mathrm{weight}\;\mathrm{of}\;\mathrm{RCA}\;\mathrm{in}\;\mathrm{nanoparticles}}{\mathrm{weight}\;\mathrm{of}\;\mathrm{RCA}\;\mathrm{NPs}}\;\times \;100\%$$

RCA NPs were suspended in ultrapure water and placed in microcuvettes. The particle size, polydispersion index (PDI), and zeta potential of RCA NPs were determined using a dynamic light scattering nanosizer (Zetasizer Nano ZS 90, Malvern, UK). The suspension was dropped onto a carbon film, dried under an infrared light, and then subjected to transmission electron microscopy (TEM, JEM-2100, JEOL, Akishima, Japan) to observe its morphology.

RCA NP stability was evaluated by dispersing them in pH 7.4 phosphate-buffered saline (PBS), simulated gastric fluid (SGF), and simulated intestinal fluid (SIF), respectively. Then, they were placed in a 37 °C incubator (KS 3000 i control, IKA, Staufen, Germany). The mean hydrodynamic diameter and PDI were measured by the nanosizer at specific time points (0, 2, 4, 6, 8, 10, 12, and 24 h).

The in vitro release of RCA from the nanoparticles was analyzed using the dialysis method [17]. Briefly, RCA and RCA NPs at a concentration of 1 mg·mL^−1^ (calculated by RCA) were dispersed in three releasing media (pH 7.4 PBS, SGF, and SIF containing 1% tween 80). They were sealed in dialysis bags and immersed in 50 mL release medium. They were then placed on a shaking bed at a constant temperature of 37 °C at 100 rpm. At predetermined time intervals (10 min, 15 min, 30 min, 45 min, 1 h, 2 h, 4 h, 8 h, 12 h, and 24 h), 1.0 mL release medium was removed and supplemented with the same volume of release medium. The drug content was determined using the HPLC method. The drug release study was conducted three times in parallel at each time point.

### 2.6. Pharmacokinetics in Rats

Twelve SD rats were randomly divided into two groups that received intragastric administration of RCA and RCA NPs at a dose of 750 mg·kg^−1^ (calculated from RCA). Blood was collected from the posterior orbital venous plexus into heparinized tubes at predefined times (0.083, 0.25, 0.5, 1, 2, 4, 6, 8, 12, 24, and 48 h post-administration). The tubes were immediately centrifuged at 13,000 rpm at 4 °C for 15 min. The serum was collected and stored at −80 °C. The concentrations of six alkaloids were detected using the UPLC-MS/MS analysis method. Chromatographic separation was performed on a Waters ACQUITY UPLC BEH C18 column (2.1 × 150 mm, 1.7 μm) at a flow rate of 0.3 mL·min^−1^. The column temperature and injection volume were 25 °C and 2 μL, respectively. The mobile phase comprised 0.1% formic acid water (mobile phase A) and acetonitrile (mobile phase B). Gradient elution was performed, as shown in Table S1. A triple quadrupole mass spectrometer equipped with atmospheric pressure electrospray ionization (APESI) was scanned in positive-ion mode and in multiple reaction monitoring (MRM) scanning mode. The ion source temperature was 350 °C, the capillary voltage was 3.2 kV, the degassing velocity was 600 L·HR-1, and the cone velocity was 50 L·HR-1. The mass spectrum parameters and selective detection ion pairs for the six alkaloids are shown in Table S2. The pharmacokinetic parameters were calculated using DAS 2.0 software.

### 2.7. Tissue Distribution Assay

The C57BL/6J mice were intragastrically administered RCA and RCA NPs at a dose of 150 mg·kg^−1^ (calculated from RCA) once daily for a continuous 4-week intervention. After the last dose was administered, the mice were anesthetized with isoflurane. Blood and major tissues were collected and processed for further analysis. Approximately 0.2 g of tissue samples were weighed accurately and homogenized with 1 mL of physiologic saline. Then, the samples were added with 100 μL IS (nuciferine, 100 ng·mL^−1^), vortexed for 2 min, and centrifuged at 13,000 rpm at 4 °C for 15 min. The supernatant was pipetted out, added to 1.2 mL acetonitrile, and vortexed for 2 min. After centrifuging at 13,000 rpm for 15 min, the acetonitrile supernatant was collected and evaporated under nitrogen gas flow. Finally, the residue was re-dissolved with 200 μL methanol and filtered through a 0.22 μm microporous membrane. The drug content was determined using the UPLC-MS/MS analysis method and normalized to tissue protein concentration.

### 2.8. Pharmacodynamics on T2DM Mice Model

#### 2.8.1. Determination of Physiological and Biochemical Indexes

The T2DM model mice were divided into four groups (10 per group): model mice group, RCA administration group (administration dosage, 150 mg·kg^−1^), RCA NPs administration group (administration dosage, 935 mg·kg^−1^), and metformin administration group (administration dosage, 200 mg·kg^−1^). The normal mouse group was set as the control. Drugs were intragastrically administered once a day for a continuous 4-week intervention. BG levels were monitored weekly. At the end of dosing, TC, TG, LDL-C, and HDL-C levels in serum and TC and TG levels in the liver were determined using biochemical kits. INS, IL-1β, IL-6, TNF-α, AST, and ALT levels in serum were determined using mouse ELISA kits. The insulin resistance index (HOMA-IR) was calculated as follows: HOMA-IR = fasting blood glucose (mmol·L^−1^) × fasting insulin (mIU·L^−1^)/22.5.

#### 2.8.2. OGTT and ITT

An oral glucose tolerance test (OGTT) was conducted after 3 weeks of treatment. The mice had free access to drinking water but were fasted overnight before sample collection. The mice were injected intraperitoneally with a glucose solution at a dose of 2 g·kg^−1^. Blood samples were collected from the terminal tail vein of mice at predefined time points (0, 15, 30, 60, 90, and 120 min), and BG was determined using a glucometer.

An insulin tolerance test (ITT) was conducted after 4 weeks of treatment. After fasting for 12 h, the mice were injected intraperitoneally with bovine insulin at a dose of 0.75 U·kg^−1^, and the remaining steps were the same as those for the OGTT. Finally, the area under the blood glucose curve (AUC) for each group of mice was calculated using the formula below:

AUC = 0.25 × (0.5 BG 0 min + BG 15 min + 1.5 BG 30 min + 2 BG 60 min + 2 BG 90 min + BG 120 min).

#### 2.8.3. Hematoxylin and Eosin (H&E) Staining

Liver tissues were collected, fixed in 4% paraformaldehyde, embedded in paraffin, and sectioned at 5 μm thickness. The paraffin sections were baked at 100 °C overnight, then dewaxed with xylenes I and II for 20 min, respectively. Then, the sections were rehydrated in an ethanol series (100, 100, 95, 80, and 70% ethanol, 5 min/time) and finally washed with ultrapure water for 5 min. Next, the sections were stained with hematoxylin and eosin for 5 min. Finally, the sections were routinely dehydrated and observed under light microscopy.

#### 2.8.4. Oil Red O Staining

Frozen livers were sectioned at 5 μm thickness and mounted onto slides. They were air-dried for 15 min at room temperature, then washed with ultrapure water and 60% isopropyl alcohol for 30 s each. The sections were then stained with 0.1% pre-warmed Oil Red O solution for 10 min. After differentiation in a 60% propylene glycol solution for 5 min, the sections were stained with hematoxylin for 30 s. Finally, the sections were observed under light microscopy.

### 2.9. Mechanism Study

#### 2.9.1. Quantitative Real-Time Polymerase Chain Reaction

The mRNA expression of proinflammatory cytokines (Il-1β, Il-6, and Tnf-α) and PI3K/AKT signal pathway-related proteins (Irs1, Pi3k, Akt, and Glut4) were detected by quantitative real-time polymerase chain reaction (qRT-PCR). Total RNA was extracted with the TRIzol Reagent (Ambion, China). Total RNA was then reverse transcribed into cDNA using the RevertAid First Strand cDNA Synthesis Kit (Thermo, Waltham, MA, USA). Real-time PCR was performed using the Power SYBR Green PCR Master Mix (Thermo, USA). Details of each primer sequence are presented in Table S3. Subsequently, gene expression was quantified using the ABI Step One Plus™ RT-PCR instrument (Thermo Fisher, USA). mRNA expression levels were calculated using the 2^−ΔΔCT^ method, and GAPDH was used as the internal reference.

#### 2.9.2. Western Blot Analysis

Total protein from liver tissue was obtained for the Western blot analysis. The liver tissues were homogenized in RIPA buffer. Protein concentration was determined using the BCA protein detection kit (Beyotime, Beijing, China). Total protein was separated by SDS-PAGE electrophoresis, transferred to a PVDF membrane blocked with 5% fat-free milk at 4 °C for 60 min, and incubated overnight with primary antibodies IL-1β (1:1000, abcam), IL-6 (1:1000, abcam), TNF-α (1:1000, abcam), IRS1 (1:1000, abcam), p-IRS1 (1:1000, Thermo Fisher Scientific), PI3K (1:2000, proteintech), p-PI3K (1:1000, Thermo Fisher Scientific), AKT (1:5000, proteintech), p-AKT (1:5000, proteintech), GLUT4 (1:2000, proteintech), and β-actin (1/10,000, proteintech). Matching horseradish peroxidase (HRP) conjugated secondary antibodies (1:5000, proteintech) were used to evaluate protein expression. The PVDF membrane was washed three times with TBST and treated with a chemiluminescence reagent. Western blot bands were quantified using Image J1.x software (NIH, Bethesda, MD, USA).

### 2.10. Statistical Analysis

SAS 8.2 software was used to analyze the experimental data. All measurement data are given as the mean ± standard deviation (SD). Student’s *t*-test analysis was used to compare data between two groups, and Tukey’s one-way analysis of variance (ANOVA) was used to compare data between multiple groups. *p* < 0.05 was considered statistically significant.

## 3. Results

### 3.1. Chemical Characterization of RCA

**Figure 1:**
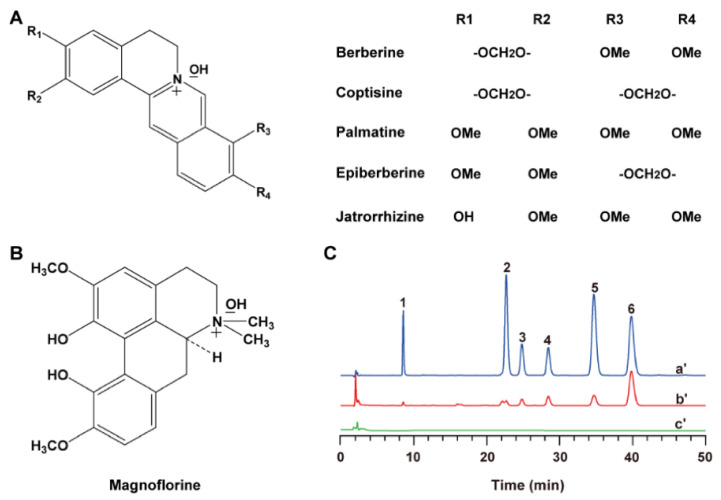
(**A**,**B**) Chemical structures of six alkaloids. (**C**) HPLC chromatograms. a’, reference substance of six alkaloids (1, magnoflorine; 2, jatrorrhizine; 3, epiberberine; 4, coptisine; 5, palmatine; 6, berberine); b’, extract solution of RCA; c’, blank solution.

Alkaloids were the main active ingredients of Rhizoma Coptidis in treating diabetes. Following extraction of total alkaloids from Rhizoma Coptidis, an HPLC determination method was established to quantify six main alkaloids in RCA (Figure 1), with berberine constituting the most abundant fraction (445.11 ± 10.79 mg·g^−1^), followed by coptisine (85.32 ± 3.46 mg·g^−1^), palmatine (44.73 ± 2.57 mg·g^−1^), jatrorrhizine (16.80 ± 1.23 mg·g^−1^), epiberberine (16.74 ± 1.44 mg·g^−1^), and magnoflorine (1.87 ± 0.38 mg·g^−1^). The proportion of six alkaloids in total alkaloids was calculated as 61.06 ± 2.85%.

### 3.2. Characterization of RCA NPs

**Figure 2:**
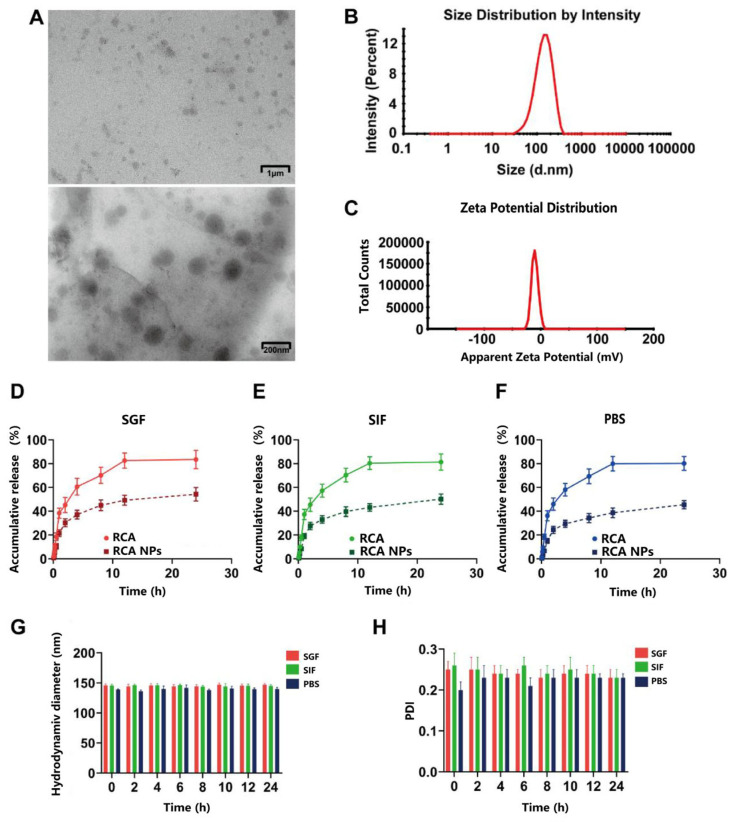
(**A**) TEM images of RCA NPs. (**B**) Particle size distribution of RCA NPs. (**C**) Zeta potential distribution of RCA NPs. (**D**–**F**) In vitro drug release curve of RCA NPs in SGF, SIF, and pH 7.4 PBS within 24 h. (**G**,**H**) Hydrodynamic diameters and PDI of the RCA NPs after co-incubating with SGF, SIF, and PBS at 37 °C for 24 h.

The RCA NP preparation process was optimized using a single-factor experiment, and the average drug loading of the optimized nanoparticles was 16.04% ± 0.22% (Table S4). The RCA NPs had a spherical shape without adhesion (Figure 2A). The average particle size was 146 ± 4 nm with a narrow size distribution (PDI = 0.248 ± 0.006) (Figure 2B), and the average zeta potential was −10.57 ± 0.31 mV (Figure 2C). As shown in Figure 2D–F, the cumulative drug release percentages of the RCA solution and RCA NPs was approximately 80% vs. 60% within 24 h, indicating that RCA NPs possessed a sustained release behavior. The particle size and PDI of RCA NPs had no obvious change after co-incubating with the release medium for 24 h (Figure 2G,H), indicating that they were stable in the gastrointestinal tract.

### 3.3. Pharmacokinetics of RCA NPs in Rats

**Figure 3:**
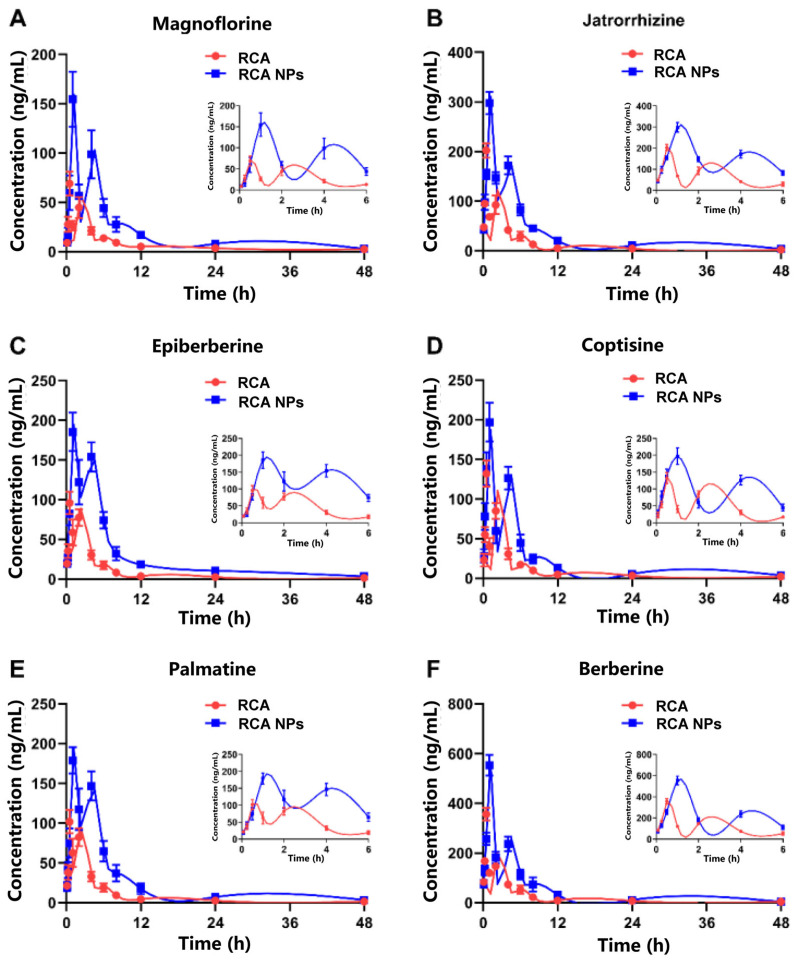
Mean plasma concentration–time curves of six alkaloids in RCA and RCA NPs: (**A**) magnoflorine; (**B**) jatrorrhizine; (**C**) epiberberine; (**D**) coptisine; (**E**) palmatine; (**F**) berberine. Data are shown as mean ± SD (*n* = 6).

A UPLC-MS/MS method was developed and validated to determine the six alkaloids in plasma. The drug concentration–time curves of the six alkaloids in rats are shown in Figure 3A–F; they shared comparable pharmacokinetic characteristics, which might be due to their similar physicochemical properties. The pharmacokinetic parameters of the six alkaloids are summarized in Table S5. Taking berberine as an example, the Cmax of RCA and RCA NPs was 356 ± 25 vs. 553 ± 41 (ng·mL^−1^), AUC0-T was 923 ± 56 vs. 2066 ± 180 (ng × h·mL^−1^), Tmax was 0.51 ± 0.03 vs. 0.99 ± 0.05 (h), and MRT0-T was 4.70 ± 0.28 vs. 5.19 ± 0.41 (h). These results demonstrate that RCA NPs had higher oral bioavailability and lower clearance in rats compared with the RCA solution.

### 3.4. Tissue Distribution of RCA NPs in Mice

**Figure 4:**
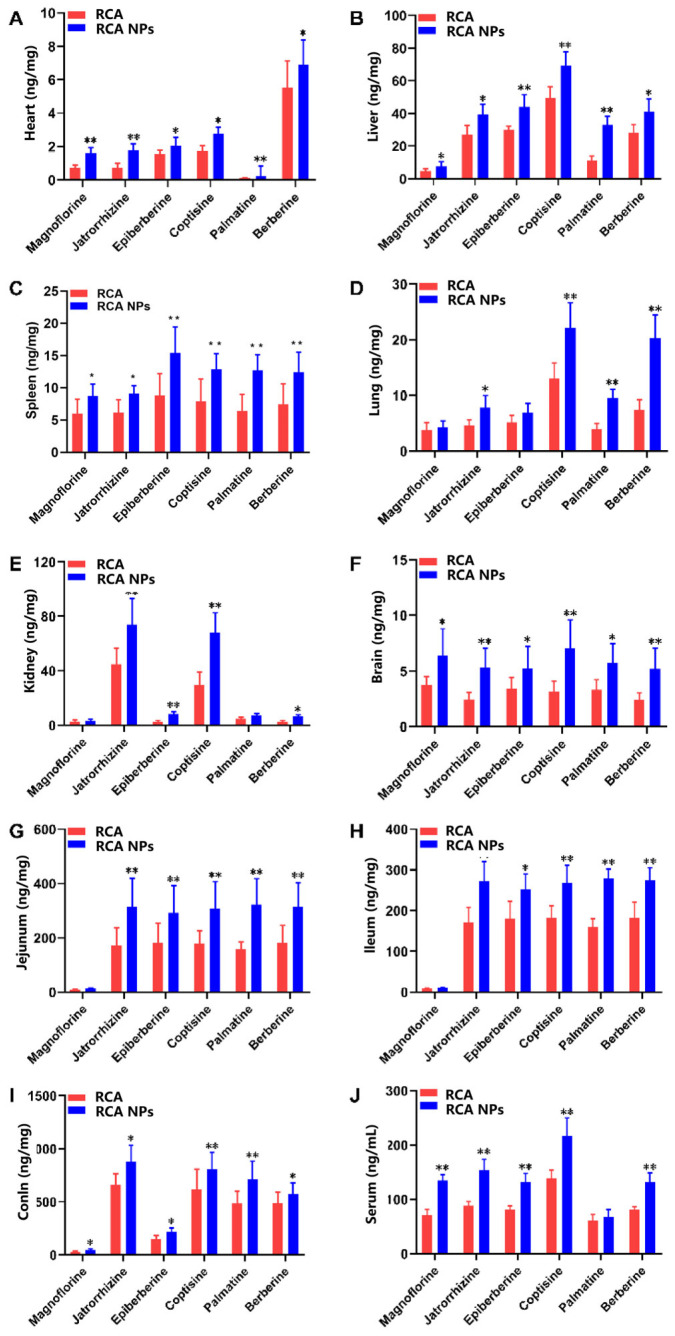
Tissue distribution of six alkaloids in RCA and RCA NPs in T2DM mice: (**A**) heart; (**B**) liver; (**C**) spleen; (**D**) lung; (**E**) kidney; (**F**) brain; (**G**) jejunum; (**H**) ileum; (**I**) colon; (**J**) serum. Data are shown as mean ± SD (*n* = 6). * *p* < 0.05, ** *p* < 0.01 vs. the RCA group.

Tissue distribution analysis showed that the six alkaloids were widely distributed in all the tissues examined (Figure 4). For both the RCA and RCA NPs groups, the drug exposure of six alkaloids in colon tissues was the highest, followed by the jejunum, ileum, serum, liver, kidney, lung, spleen, brain, and heart. Due to the better bioavailability of the RCA NPs group, the contents of the six alkaloids in all tissues were higher than in the RCA group. Moreover, nanoparticles were easily captured by the reticuloendothelial system, resulting in a high drug content in the liver of the RCA NPs group. Because the liver is a vital organ for glycogen synthesis, glycolysis, and gluconeogenesis, it could be inferred that the hypoglycemic effect of RCA NPs would be better than that of RCA.

### 3.5. RCA NPs Improved Glucose and Lipid Metabolism Disorder in T2DM Mice

**Figure 5:**
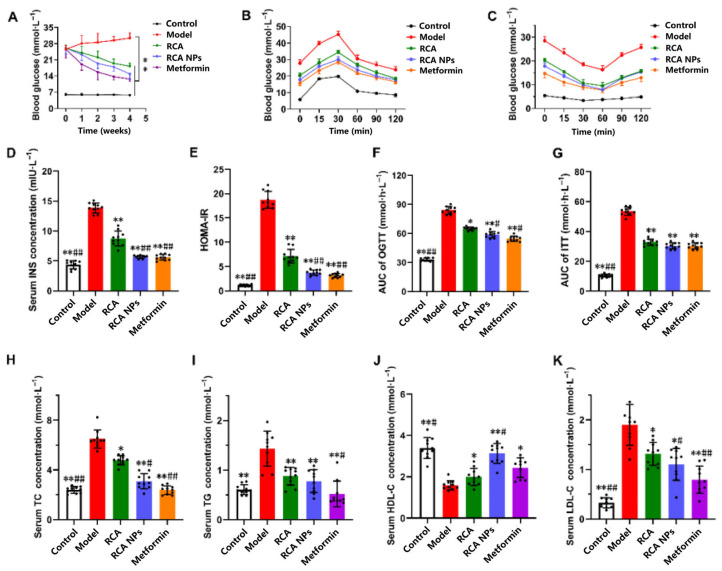
Hypoglycemic and hypolipidemic effects of RCA NPs in T2DM mice. (**A**) Blood glucose. (**B**) Oral glucose tolerance test. Glucose levels at 0, 15, 30, 60, 90, and 120 min after intraperitoneal injection with glucose. (**C**) Insulin tolerance test. Glucose levels at 0, 15, 30, 60, 90, and 120 min after intraperitoneal injection with bovine insulin. (**D**) Serum insulin concentration. (**E**) HOMA-IR. (**F**) AUC of the OGTT curve. (**G**) AUC of the ITT curve. (**H**) Serum TC concentration. (**I**) Serum TG concentration. (**J**) Serum HDL-C concentration. (**K**) Serum LDL-C concentration. Data are shown as mean ± SD (*n* = 10). * *p* < 0.05, ** *p* < 0.01 vs. the model group. # *p* < 0.05, ## *p* < 0.01 vs. the RCA group.

A T2DM mouse model was established to investigate anti-diabetes effects. As shown in Figure 5A, BG levels in model mice were significantly higher than those in normal mice (*p* < 0.05), demonstrating that the modeling method was effective. BG levels improved significantly after drug intervention. In the OGTT test (Figure 5B,F), compared with an AUC value of 85.90 mmol·h·L^−1^ in the model group, those values in the RCA, RCA NPs, and metformin groups were 69.54, 61.36, and 55.22 mmol·h·L^−1^, which were reduced by 19.04%, 28.57% and 35.72%, respectively. In the ITT test (Figure 5C,G), AUC values decreased by 38.10%, 42.86%, and 45.24%, respectively. In addition, the serum insulin level and HOMA-IR in the RCA NPs group decreased by more than 2-fold compared with those in the model group (Figure 5D,E). Hypolipidemic effect evaluation found that the serum TC, TG and LDL-C contents of RCA NPs-treated mice were significantly decreased, while HDL-C content was significantly increased compared with model mice (Figure 5H–K). These results demonstrate that both RCA and RCA NPs can reduce blood glucose and blood lipid levels, improve glucose and lipid metabolism, and improve insulin sensitivity in T2DM mice. Moreover, RCA NPs can significantly enhance the hypoglycemic and hypolipidemic efficacy of RCA.

### 3.6. RCA NPs Ameliorated Liver Function in T2DM Mice

**Figure 6:**
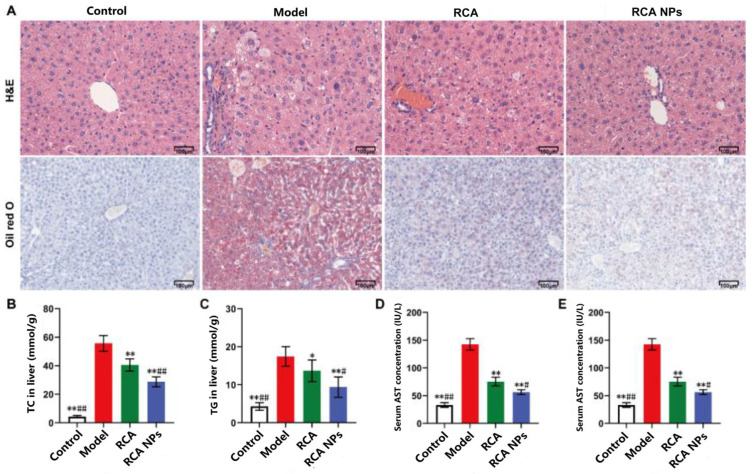
Effects of RCA NPs on liver function in T2DM mice. (**A**) H&E and Oil Red O staining of liver tissues (×200 magnification; scale bar: 100 μm). (**B**) Liver TC concentration. (**C**) Liver TG concentration. (**D**) Liver AST concentration. (**E**) Liver ALT concentration. Data are shown as mean ± SD (*n* = 10). * *p* < 0.05, ** *p* < 0.01 vs. the model group. # *p* < 0.05, ## *p* < 0.01 vs. the RCA group.

The liver plays a crucial role in regulating blood glucose and lipid homeostasis [18]. To evaluate the effects of RCA NPs on liver function under the T2DM state, liver tissue morphology and lipid droplet contents in liver tissue were observed by H&E and Oil Red O staining, respectively. Figure 6A shows that liver cells in the model group had severe vacuolar degeneration and stored a large number of lipid metabolites. These symptoms were alleviated after treatment with RCA and RCA NPs, suggesting that RCA and RCA NPs could inhibit liver lipid deposition and reduce the degree of liver tissue injury. Compared with the RCA group, TC and TG levels in the RCA NPs group decreased by over 30%, and AST and ALT in serum decreased by over 50% (Figure 6B–E), suggesting that RCA NPs could enhance the liver protective effect of RCA.

### 3.7. RCA NPs Reduced Inflammation Levels in Liver of T2DM Mice

**Figure 7:**
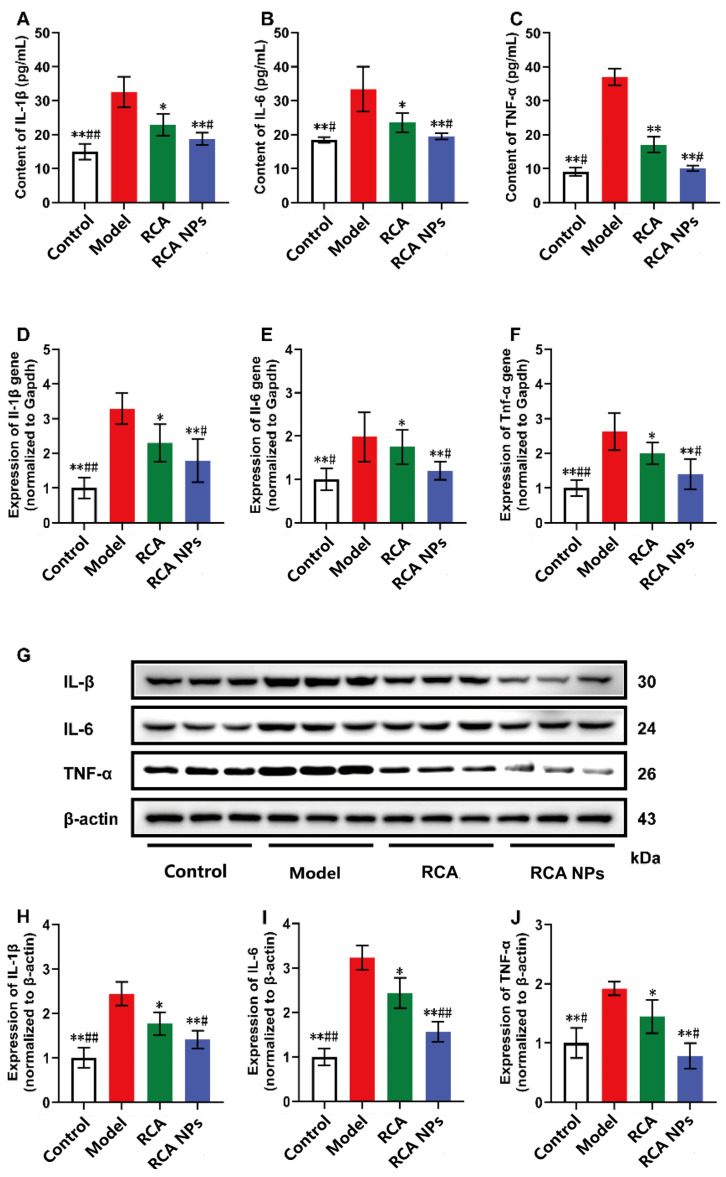
Anti-inflammatory effect of RCA NPs. (**A**–**C**) Serum IL-1β, IL-6, and TNF-α concentrations. (**D**–**F**) Relative expressions of Il-1β, Il-6, and Tnf-α mRNA in liver. (**G**) Western blotting assays of IL-1β, IL-6, and TNF-α in liver tissues. (**H**–**J**) Relative expressions of IL-1β, IL-6, and TNF-α in liver tissues. Data are shown as mean ± SD (*n* = 10). * *p* < 0.05, ** *p* < 0.01 vs. the model group. # *p* < 0.05, ## *p* < 0.01 vs. the RCA group.

Studies have shown that chronic inflammation is associated with T2DM onset and development [19]. The T2DM model mice exhibited elevated serum levels of proinflammatory cytokines (TNF-α, IL-1β, and IL-6) compared with the normal group (Figure 7A–C). TNF-α, IL-1β, and IL-6 gene and protein expression in liver tissues were further detected. Tnf-α, Il-1β, and Il-6 gene expression were also significantly upregulated in the liver tissues of T2DM mice (Figure 7D–F), consistent with the quantitative analysis results of WB (Figure 7G–J). After treatment with RCA and RCA NPs, mRNA and protein expression was significantly down-regulated, and the RCA NPs group had a better therapeutic effect than the RCA group. These results demonstrate that RCA NPs also had anti-inflammatory effects.

### 3.8. RCA NPs Promoted the Expression of PI3K/AKT Signaling Pathway

**Figure 8:**
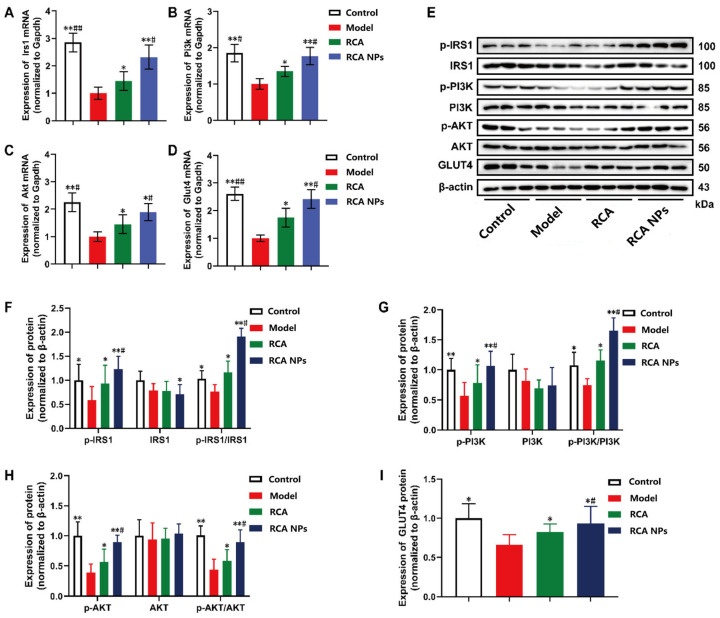
Effects of RCA NPs on PI3K/AKT signaling pathway in liver tissue of T2DM mice. (**A**–**D**) Relative expressions of Irs1, Pi3k, Akt, and Glut4 mRNA. (**E**) Western blotting assays of p-IRS1, IRS1, p-PI3K, PI3K, p-AKT, AKT, and GLUT4 in liver tissues. (**F**–**I**) Relative expression of p-IRS1, IRS1, p-PI3K, PI3K, p-AKT, AKT, and GLUT4 in liver tissues. Data are shown as mean ± SD (*n* = 3). * *p* < 0.05, ** *p* < 0.01 vs. the model group. # *p* < 0.05, ## *p* < 0.01 vs. the RCA group.

PI3K/AKT is a classical insulin signaling pathway for regulating glucose uptake [20]. Figure 8A–D shows that compared with normal mice, the mRNA expression levels of Irs1, Pi3k, Akt, and Glut4 were significantly decreased in T2DM mice. Following treatment with RCA and RCA NPs, the expression of these four genes was significantly increased. WB results also showed that RCA and RCA NPs significantly upregulated IRS1, PI3K, Akt, and GLUT4 protein levels in liver tissues (Figure 8E–I), consistent with gene expression. Moreover, protein phosphorylation levels were elevated, indicating enhanced protein activity. These results suggest that RCA NPs could effectively activate the IRS1/PI3K/AKT/GLUT4 signaling pathway, thereby increasing glucose uptake in liver tissue.

## 4. Discussion

In Chinese herbal formulas, Rhizoma Coptidis has been widely used to treat diabetes. RC is commonly used in combination with other herbs to form a compound formula. The main applications are decoctions, pills, capsules, etc. [21]. Extensive studies have uncovered that alkaloids are the main chemical components and therapeutic components. In particular, berberine has been used in the clinic as a natural medicine for treating gastroenteritis and as an adjuvant therapy for treating diabetes [22,23]. As a star natural compound, various nanocarriers have been designed to deliver berberine for treating different diseases such as diabetes, ulcerative colitis, atherosclerosis, and cancer [24,25,26]. In particular, in recent years, berberine has been widely used to construct carrier-free nanodrugs via a self-assembly mechanism. For example, berberine and rhein could self-assemble into nanoparticles via hydrogen bonding, π–π interaction, and electrostatic interaction, which significantly increased antibacterial efficacy due to a synergistic effect [27]. To the best of our knowledge, RCA-loaded nanomedicine has not been reported before. In the present study, degradable PLGA was employed to prepare polymer-based nanoparticles for oral delivery of RCA. By encapsulating RCA in nanoparticles, they benefited from increased solubility and sustained drug release. In addition, PLGA degraded slowly in vivo, which avoided burst release in the gastrointestinal tract.

RCA NPs showed significantly higher distribution of six alkaloids in plasma and various tissues compared with the RCA group, indicating that nanoparticles could be better absorbed into circulation. The transport mechanism of PLGA-based nanoparticles mainly includes M cell-mediated uptake, endocytosis by intestinal epithelial cells, mucus layer penetration, and bio-adhesion [28]. After nanoparticles entered the systemic circulation, they were readily captured by the reticuloendothelial system, which might explain the higher drug concentration in the liver than in other tissues [29]. Interestingly, multiple peaks appeared in the drug–time curves, possibly due to an enterohepatic circulation effect [30].

The T2DM mouse model was induced by a high-fat diet, which also resulted in fatty liver. This finding was consistent with clinical observations that T2DM is often accompanied by fatty liver. Interestingly, RCA and RCA NPs showed hypoglycemic, hypolipidemic, and anti-inflammatory effects, which could be attributed to the multi-pharmacological effects of RCA. Previous mechanism studies on Rhizoma Coptidis have mainly focused on its aqueous extracts and the monomeric compound berberine, whereas research into its total alkaloids has been relatively limited. This study revealed that berberine exerts its therapeutic effects through the regulation of multiple signaling pathways, including AMPK/mTOR, TLR4/NF-κB, and PI3K/AKT [31]. In addition, the gut microbiome is an important factor in treating diabetes that is increasingly attracting the attention of researchers [32]. In the present study, the PI3K/AKT-related signaling pathways were selected to illustrate the mechanism of action because PI3K/AKT are co-signaling molecules in diabetes, obesity, and inflammation [33]. Though changes in expression of IRS1/PI3K/AKT/GLUT4 were confirmed by the protein and gene detection, further correlative study need to illustrate the link between the drug and the pathway through pathway inhibition (e.g., PI3K inhibitors) or genetic manipulation experiment.

## 5. Conclusions

We prepared oral RCA NPs, which were found to be 145 nm nanospheres with a sustained drug release profile. The solubility and bioavailability of RCA were greatly increased due to drug encapsulation and enhanced blood concentration. RCA NPs preferentially accumulated in liver tissues after entering systemic circulation. Compared with RCA, RCA NPs not only had better hypoglycemic and hypolipidemic effects but also better liver protective and anti-inflammatory effects in T2DM model mice. On the one hand, high drug concentrations in blood and liver tissues might directly result in a superior therapeutic effect of RCA NPs. On the other hand, RCA NPs strengthen the expression and activity of IRS1/PI3K/AKT/GLUT4, thus increasing insulin sensitivity and glucose uptake. Therefore, this study provides an efficient strategy for the oral delivery of RCA and anti-diabetic therapy, which holds promise for future clinical translation.

## Data Availability

Data presented in this study is contained within the article and Supplementary Material. Further inquiries can be directed to the corresponding author.

## Supplementary Materials

The following supporting information can be downloaded at: https://www.mdpi.com/article/10.3390/pharmaceutics18030349/s1, Table S1: The gradient elution procedure of UPLC-MS/MS. Table S2: The optimized mass spectrometry (MS) parameters of the six alkaloids and internal standard (IS). Table S3: Primer sequences of genes in liver tissues of T2DM mice. Table S4: Single factor analysis of the RCA NPs preparation. Table S5: Pharmacokinetic parameters of 6 alkaloids in rat plasma.
