# Supplementary material for: Oral Rhizoma Coptis Alkaloids Nanoparticle for Treating Diabetes Through Regulating PI3K/Akt Pathways
Source: Pharmaceutics. 2026 Mar 11;18(3):349. doi: 10.3390/pharmaceutics18030349 (PMC13029669; doi:10.3390/pharmaceutics18030349)
Supplement: Supplementary file 1 [file pharmaceutics-18-00349-s001.zip › pharmaceutics-4150977-supplementary.pdf]

Supplementary Material

Oral Rhizoma Coptis Alkaloid Nanoparticles for Treating Diabetes Through Regulating PI3K/Akt Pathways

Yuejiao Liu [1], Mengyuan Zhu [2], Qiaoqiao Su [2], Maofeng Liu [2], Zhenyu Zhao [1,*] and Pengkai Ma [2,*]

[1] NHC Key Lab of Hormones and Development and Tianjin Key Lab of Metabolic Diseases, Tianjin Medical University Chu Hsien-I Memorial Hospital & Institute of Endocrinology, Tianjin 300000, China

[2] School of Chinese Materia Medica, Beijing University of Chinese Medicine, Beijing 100000, China

* Correspondence: zhaozhenyu0858@163.com (Z.Z.); mapengkai1990@126.cn (P.M.)

Table S1. The gradient elution procedure of UPLC-MS/MS

Time (min)	Ratio of mobile phase B (%)
0-1	10-20
1-3	20-22.5
3-3.5	22.5-32.5
3.5-4.5	32.5-45
4.5-5	45-90
5-5.51	90-10
8.0	0

Table S2. The optimized mass spectrometry (MS) parameters of the six alkaloids and internal standard (IS)

Components	Chemical formula	Q1（M/Z）	Q3（M/Z）	CV	Collision energy
Magnoflorine	$C_{20}H_{24}NO_4$	342.20	265.22	16	24
Jatrorrhizine	$C_{20}H_{20}NO_4$	337.77	322.20	12	30
Coptisine	$C_{19}H_{15}NO_5$	320.00	292.17	96	28
Palmatine	$C_{21}H_{23}NO_4$	354.08	338.89	12	20
Epiberberine	$C_{20}H_{18}NO_4$	337.92	322.48	4	24
Berberine	$C_{20}H_{18}NO_4$	337.92	322.48	4	24
Nuciferine	$C_{19}H_{21}NO_2$	296.25	265.23	38	12

Table S3. Primer sequences of genes in liver tissues of T2DM mice

Primer	Forward	Reverse
Il-1β	CAACCAACAAGTGATATTCTCCATG	GATCCACACTCTCCAGCTGCA
Tnf-α	CACAGAAAGCATGATCCGCG	ACTGATGAGAGGGAGGCCAT
Il-6	AGCCAGAGTCCTTCAGAGAGA	GGATGGTCTTGGTCCTTAGCC
Irs1	ATCACTCAGGGCAAGCTCTT	CCCAGGAGATCTCGGAAGTC
Akt	GCAAGGAGGGGATCAAGGAC	CGGCCACACATCATCTCGTA
Pi3k	GGGTAGAATTGGCTCCATTGG	GCTATCTCATGGCGACAAGCT
Glut4	CATTCCCTGGTTCATTGTGG	GAAGACGTAAGGACCCATAGC
Gapdh	CTCCCACTCTTCCACCTTCG	TAGGGCCTCTCTTGCTCAGT

Table S4. Single factor analysis of the RCA NPs preparation

Factor		Level	EE (%)	Size (nm)	PDI
drug to carrier mass ratio (mg/mg)		1/4	5.79	207.83	0.331
		1/2	8.42	202.97	0.387
		1/1	6.03	231.60	0.316
ratio of organic phase to water phase (v/v)		1/2	9.34	219.43	0.384
		1/5	11.73	196.36	0.336
		1/10	6.35	216.76	0.247
types and dosages of emulsifiers	SDC (%)	0.1	9.34	219.43	0.384
		0.5	11.47	140.77	0.378
		1	10.13	192.90	0.388
	PVA (%)	0.1	9.39	183.47	0.294
		0.5	7.77	153.13	0.401
		1	3.36	203.20	0.747
high speed homogenization time (min)		30	15.16	146.30	0.317
		60	7.15	170.10	0.305
		120	10.49	395.63	0.548
high speed homogenization speed (rpm)		12000	10.33	164.23	0.540
		21000	11.41	154.17	0.303
		30000	15.16	146.30	0.317
homogeneous pressure (psi)		5000	7.32	310.93	0.435
		10000	9.56	207.23	0.387
		15000	10.87	175.96	0.277
number of homogeneous cycles		5	10.87	175.96	0.277
		10	15.22	170.27	0.273
		15	9.87	182.86	0.292
molecular weight of polymer (Da)		15000	13.30	185.72	0.478
		33000	9.87	480.64	0.486
		55000	11.74	759.40	0.632

Table S5. Pharmacokinetic parameters of 6 alkaloids in rat plasma

Component	Pharmacokinetic parameters	RCA	RCA NPs
Magnoflorine	T_{max}（h）	0.51 ± 0.02	1.03 ± 0.06
	C_{max}（ng·mL^{-1}）	68.97 ± 12.15	154.17 ± 27.89
	$T_{1/2}$（h）	5.78 ± 2.72	6.16 ± 2.07
	$AUC_{0\text{-}t}$（ng × h·mL^{-1}）	277.50 ± 18.10	779.13 ± 50.67
	$AUC_{0\text{-}\infty}$（ng × h·mL^{-1}）	290.88 ± 20.92	829.86 ± 88.35
	$MRT_{0\text{-}t}$（h）	6.14 ± 0.65	6.45 ± 0.59
	$MRT_{0\text{-}\infty}$（h）	8..84 ± 0.38	8.79 ± 1.04
Jatrorrhizine	T_{max}（h）	0.50 ± 0.01	1.01 ± 0.05
	C_{max}（ng·mL^{-1}）	202.37 ± 14.47	298.17 ± 22.60
	$T_{1/2}$（h）	3.18 ± 1.63	3.45 ± 1.89
	$AUC_{0\text{-}t}$（ng × h·mL^{-1}）	537.95 ± 42.92	1396.39 ± 108.82
	$AUC_{0\text{-}\infty}$（ng × h·mL^{-1}）	545.30 ± 53.07	1415.39 ± 116.86
	$MRT_{0\text{-}t}$（h）	4.64 ± 0.27	5.45 ± 0.38
	$MRT_{0\text{-}\infty}$（h）	5.66 ± 0.54	6.48 ± 0.82
Epiberberine	T_{max}（h）	0.50 ± 0.02	1.02 ± 0.04
	C_{max}（ng·mL^{-1}）	96.56 ± 13.59	186.56 ± 23.10
	$T_{1/2}$（h）	2.94 ± 0.87	3.67 ± 0.64
	$AUC_{0\text{-}t}$（ng × h·mL^{-1}）	369.78 ± 24.52	1124.47 ± 51.00
	$AUC_{0\text{-}\infty}$（ng × h·mL^{-1}）	372.25 ± 27.57	1148.36 ± 83.56
	$MRT_{0\text{-}t}$（h）	4.84 ± 0.46	5.96 ± 0.33
	$MRT_{0\text{-}\infty}$（h）	5.24 ± 0.64	7.31 ± 1.07
Coptisine	T_{max}（h）	0.49 ± 0.03	1.00 ± 0.07
	C_{max}（ng·mL^{-1}）	132.20 ± 16.36	197.00 ± 24.54
	$T_{1/2}$（h）	4.60 ± 2.86	5.04 ± 2.55
	$AUC_{0\text{-}t}$（ng × h·mL^{-1}）	403.42 ± 31.95	863.40 ± 51.88
	$AUC_{0\text{-}\infty}$（ng × h·mL^{-1}）	419.17 ± 47.77	890.94 ± 54.74
	$MRT_{0\text{-}t}$（h）	4.78 ± 0.18	5.29 ± 0.24
	$MRT_{0\text{-}\infty}$（h）	6.39 ± 1.42	6.53 ± 0.74
Palmatine	T_{max}（h）	0.50 ± 0.03	1.01 ± 0.06
	C_{max}（ng·mL^{-1}）	102.58 ± 14.30	181.43 ± 13.77
	$T_{1/2}$（h）	2.74 ± 0.37	4.38 ± 2.65
	$AUC_{0\text{-}t}$（ng × h·mL^{-1}）	403.58 ± 33.08	1068.90 ± 111.51
	$AUC_{0\text{-}\infty}$（ng × h·mL^{-1}）	404.56 ± 33.57	1101.25 ± 138.77
	$MRT_{0\text{-}t}$（h）	4.57 ± 0.59	5.65 ± 0.60
	$MRT_{0\text{-}\infty}$（h）	5.34 ± 0.88	6.86 ± 1.41
Berberine	T_{max}（h）	0.51 ± 0.03	0.99 ± 0.05
	C_{max}（ng·mL^{-1}）	356.17 ± 25.46	553.43 ± 41.29
	$T_{1/2}$（h）	3.18 ± 1.63	3.86 ± 1.39
	$AUC_{0\text{-}t}$（ng × h·mL^{-1}）	923.04 ± 56.55	2065.89 ± 180.02
	$AUC_{0\text{-}\infty}$（ng × h·mL^{-1}）	935.97 ± 60.00	2098.14 ± 201.99
	$MRT_{0\text{-}t}$（h）	4.70± 0.28	5.19 ± 0.41
	$MRT_{0\text{-}\infty}$（h）	5.77 ± 0.69	5.98 ± 0.62
